# Effects of Substituting Sweet Sorghum for Corn Silage in the Diet on the Growth Performance, Meat Quality, and Rumen Microorganisms of Boer Goats in China

**DOI:** 10.3390/ani15101492

**Published:** 2025-05-21

**Authors:** Shuyang Wang, Fangzhu Guo, Yuchen Wang, Miaoyin Dong, Junkai Wang, Guoqing Xiao

**Affiliations:** 1 Institute of Modern Physics, Chinese Academy of Sciences, Lanzhou 730000, Chinawangyuchen@shcc.edu.cn (Y.W.); dmy369maile@163.com (M.D.);; 2 University of Chinese Academy of Sciences, Beijing 100049, China; 3 Department of Customs Inspection and Quarantine, Shanghai Customs College, Shanghai 201204, China; 4 College of Life Science and Technology, Gansu Agricultural University, Lanzhou 730070, China

**Keywords:** sweet sorghum silage, corn silage, microbial community, goat, meat performance

## Abstract

Due to its superior drought tolerance, high biomass yield, and stress resistance, sweet sorghum (*Sorghum bicolor* L.) has emerged as an ideal candidate for sustainable forage production in arid, semi-arid, and mildly saline–alkaline regions. This study aimed to evaluate the effects of replacing corn silage (CS) with either forage sorghum silage (FSS) or sugar sweet sorghum silage (SSS) on goat meat quality, the rumen microbial community, and meat composition. Thirty 3-month-old Boer goats (average body weight: 13.44 ± 1.67 kg) were randomly assigned to five dietary treatments; the control group contained 50% corn silage (CON), while the experimental groups contained 50% FSS (group I), 70% FSS (group II), 50% SSS (group III), or 70% SSS (group V), with each group receiving the same concentrate diet but different roughage sources. The results showed that compared to the CON group (50% CS), the experimental groups had a significantly increased average daily weight gain (ADG) (*p* < 0.05) and slaughter rate (SR). It is noteworthy that group III (50% SSS) showed a significant increase of 12.4% in SR (*p* < 0.01). Analysis of the silage characteristics and changes in the rumen microbial community revealed that feeding SSS and FSS increased the relative abundance of *Ruminococcus* in the rumen, further enhanced the degradation and conversion of silage neutral detergent fiber (NDF), and promoted the synthesis of fatty acids and amino acids. Specifically, FSS significantly increased the amino acid content in the meat, while SSS effectively improved the crude protein (CP) and crude fat (CF) contents. In conclusion, replacing 50% CS with FSS or SSS can effectively improve the meat quality and growth performance of Boer goats.

## Simple Summary

Replacing corn silage with sweet sorghum silage, whether derived from sugar or forage sweet sorghum, presents a sustainable solution for livestock production. Sweet sorghum requires less water and can grow efficiently in poor soils, reducing competition with food crops for water and land. This study demonstrates that substituting 50% of corn silage with sweet sorghum silage leads to increased goat growth. Additionally, it improves the meat quality by enhancing the crude protein content, crude fat, and amino acids associated with flavor. These benefits are linked to changes in the rumen microbiota, particularly an increase in *Ruminococcus*, which aids in nutrient breakdown and utilization. This study offers a practical strategy for forage development in arid, mildly saline–alkaline, and infertile soil areas while producing high-quality meat.

## 1. Introduction

In arid, semi-arid, and mildly saline–alkaline areas, sweet sorghum, as a drought-tolerant and stress-resistant crop, has significant advantages. Compared to corn, sweet sorghum is more suitable for growing in arid and semi-arid conditions due to its slower leaf and stalk wilting, recovery after drought events, and lower irrigation requirements [1]. Therefore, sweet sorghum has become an ideal fodder crop in areas with limited water resources [2]. Sweet sorghum can be divided into sugar sweet sorghum and forage sweet sorghum. Sugar sweet sorghum accumulates more sugar, making it suitable for extracting sugar and producing other by-products. The high sugar content is also more easily digested and absorbed by feeding animals, which helps in reducing the severity of rumen acidosis [3]. Forage sweet sorghum is relatively low in sugar content compared to sugar sweet sorghum, but its very short growing cycle (about 60 days) and high biomass production under drought conditions, yielding up to 10–20 tons of dry matter (DM) per hectare [4,5], make it an ideal livestock feed.

The main forages utilized by livestock in the western region of China are common sweet sorghum and corn, which are available at reasonable costs but with reduced nutritive value (energy and crude protein concentration) and digestibility [6]. Sweet sorghum is comparable in nutritional value to corn [7], and it contains higher water-soluble carbohydrates compared to corn [8]. Additionally, sweet sorghum can be harvested twice a year, while corn is limited to a single harvest [9]. Therefore, sweet sorghum not only provides adequate nutrition for livestock but can also be fermented into silage to improve digestibility and nutritional value, making it a high-quality feed source for ruminants such as goats.

Sweet sorghum silage (SSS) is under consideration as a suitable feed for goat meat producers. Some researchers showed that compared to corn, sweet sorghum is more favorable to be a forage source for goats in that it contains moderate levels of crude protein, higher gross energy, and has great digestibility. Replacing corn silage with high-sugar sorghum silage in the diet of dairy cows without additional grain supplementation has no negative effect on feed intake, milk production, nitrogen utilization, and rumen fermentation [9]. Sweet sorghum silage also helps maintain a stable rumen environment in sheep [10]. It is early maturing, reaching a mean maximum weight of ≈62 kg at 3.5 years of age on a natural pasture under extensive grazing conditions [11]. The Boer goat is a remarkable small-stock ruminant that possesses distinctive qualities enabling it to excel as an efficient red meat producer [12].

Thus far, relatively few studies have reported the substituting SS for corn silage feeding on goat meat performance of Boer goats. Therefore, this study aims to evaluate the effects of replacing CS with varying proportions of SSS or FSS on the growth performance, meat quality, and rumen microbiota of Boer goats.

## 2. Materials and Methods

### 2.1. Harvesting and Ensiling of Sweet Sorghum

Forage sweet sorghum (FS, hybrid BJ6003) and sugar sweet sorghum (SS, hybrid BJ6002) samples were obtained during the growing season on 15 October 2022 from the experimental field of Wuwei Test Station, Gansu Province, China (37°43′ N latitude, 102°35′ E longitude; 2200–2400 m above sea level). The FS sample was chopped into approximately 1.0–2.0 cm particles using a forage harvester (9Z-1.8A, Xinshun, Maomin, China). The SS sample was obtained from the residue of sugar sorghum after juice extraction. The FS and SS materials were mixed with sweet sorghum silage inoculant (supplied by Institute of Modern Physics, Chinese Academy of Sciences, Lanzhou, China; the additive consists mainly of lactic acid bacteria as well as cellulase) at a rate of 1 mL/kg of fresh weight. Supplementary water was added to adjust the moisture content of FS and SS to 75%, and after thorough mixing, 50 kg was packed into polyethylene silage bags (50 cm diameter × 50 cm height) and ensiled for 3 months. The corn silage (CS) used in this study was purchased from the Agricultural Cooperative of Huangyang Township, Wuwei, Gansu, China.

Samples were collected using the conical and quartering methods and thoroughly mixed. Fresh silage samples were extracted with sterile distilled water at a ratio of 1:10, and the organic acid content was determined using liquid chromatography. The remaining silage samples were dried at 65 °C for 48 h for the determination of dry matter (DM), water-soluble carbohydrates (WSC), crude protein (CP), neutral detergent fiber (NDF), acid detergent fiber (ADF), crude fiber, and ash content. The chemical compositions of the silages (FSS, SSS, and CS) are presented in Table 1.

### 2.2. Microbial Community Analysis of Silage

Microbial communities were analyzed in the silage samples. The silage samples (100 g) were added to 500 mL of sterilized phosphate-buffered saline (pH 7.4) and sonicated in an Ultrasonic cleaning bath (VGT-2013QTD, Gute, Meizhou, China) for 10 min at room temperature. After sonication, the water extracts were subjected to centrifugation at 12,000× *g* for 15 min. The microorganisms on the surface of the collected silage were separately extracted from the total microbial DNA using the TIANamp Bacterial DNA Isolation Kit (DP302-02, Tiangen, Beijing, China) according to the method provided by the manufacturer. Then, the extracted DNA samples were used for amplifying the V3–V4 hypervariable region of the 16S rRNA gene with a universal primer (forward, 5′-ACTCCTACGGGAGGCAGCA-3′; reverse, 5′-GGACTACHVGGGTWTCTAAT-3′).

### 2.3. Microbial Community Analysis of the Rumen

The day before the end of feeding, rumen fluid was collected from the oral cavity using a rumen tube, and the collected rumen fluid was filtered through four layers of gauze. The microorganisms of rumen were separately extracted from the total microbial DNA using the TIANamp Bacterial DNA Isolation Kit (DP302-02, Tiangen, Beijing, China) according to the method provided by the manufacturer. Then, the extracted DNA samples were used for amplifying the V3-V4 hypervariable region of the 16S rRNA gene with a universal primer (forward, 5′-ACTCCTACGGGAGGCAGCA-3′; reverse, 5′-GGACTACHVGGGTWTCTAAT-3′).

### 2.4. Illumina Miseq Sequencing and Data Analysis

The amplicon libraries were sequenced via paired-end sequencing on an Illumina Miseq platform at the Biomaker Company Co., Ltd. (Beijing, China). For improving the quality of the original data, Trimmomatic (v.0.33) software was used to discard the reads containing barcode or primer errors, and UCHIME (v.4.2) was used to identify and remove the chimeric sequences. After filtering process, the effective tags (at least 200 bp long) were clustered into operational taxonomic units (OTUs) with a threshold of 97% sequence similarity (QIIME v.1.8.0). The OTU file was used to evaluate the alpha (Mothur v.1.30) and beta diversities (QIIME v.1.8.0).

### 2.5. Experimental Design and Diets

The study was conducted at Tuopu grass industry Co., Ltd., Wuwei, China. Thirty Boer goats ♂ (initial age = 3.0 months; initial body weight, BW = 13.44 ± 1.67 kg) were randomly classified into five groups with different formulas of dietary treatments, and each group consisted of six goats in three adjacent open-sided pens, with three parallel experiments. Animal experimental procedures have been carried out in accordance with the “Experimental animal management regulations” of China. The diet compositions for the five treatments are presented in Table 2. The concentrate diet was mixed well with the ingredient at a ratio of 1:3, where the roughage ingredient treatments for each group were as follows: group I: roughages containing 50% FSS; group II: roughages containing 70% FSS; group III: roughages containing 50% SSS; group IV: roughages containing 70% SSS; and CON: roughages containing 50% CS. The concentrates were also provided by Tuopu grass industry Co., Ltd., Wuwei, China. Forages were offered twice daily at 09:00 and 18:30, and water was offered ad libitum throughout the study duration. The study lasted for 105 d, which contained a 15 d adaptation period and a 90 d period for data collection. The weights and average daily gain of individual goats were recorded.

The experimental Boer goats were slaughtered in accordance with the conventional method at the end of experiments. A 500 g meat sample from the forelegs and back legs of each goat were taken, packed immediately with vacuum packaging bags, and transported with ice boxes to the laboratory for analysis. Slaughter rate (SR) were calculated using the following equation:

(1) $$Slaughter\;rate=\frac{C}{W}\times 100\%$$

where C is the weight of the carcass after slaughter, and W is the weight of the goat before slaughter.

### 2.6. Laboratory Analysis

Samples of FSS and SSS in each group were dried in a forced-air oven at 65 °C for 48 h to a constant weight (dry matter, DM) (International Organization for Standardization (ISO) 6496) and ground to pass a 1 mm sieve for analysis (Arthur H. Thomas Co., Chadds Ford, PA, USA). pH determination was carried out using a calibrated portable pH meter (pH meter model PB-10, Sartorius Inc. Beijing, China). Neutral detergent fiber (NDF) analysis was performed according to procedures described in a modified method of [13] with the use of heat stable amylase (ISO 16472). Acid detergent fiber (ADF) was determined via boiling with ADF reagent and expressed without residual ash (ISO 13906:2008). Both NDF and ADF were expressed with residual ash by using the fiber analyzer apparatus (Shengtai ST-04B, Shandong, China) [13]. Ash was determined via incineration at 550 °C for 4 h (ISO 5984). Crude protein was calculated as N × 6.25, where N is the nitrogen content, which was determined using a Kjeldahl apparatus (model ST-04C, Shengtai, Ltd., Jinan, China). Lactic acid, acetic acid, propionic acid, and butyric acid were measured using HPLC (2690 Waters, Ltd., Milford, MA, USA) according to the procedures of [14].

All experimental goats were sent to the Wuwei meat processing plant for slaughter at the end of the experiment. Goat meat samples were collected from each goat and analyzed immediately for their pH, initial moisture, ash, crude fat, protein (N × 6.38), 18 types of structural amino acids, and fatty acids. Crude fat was analyzed using Soxhlet extraction. Eighteen types of structural amino acids were analyzed using an amino acid analyzer (835-0200; Hitachi, Ltd. Tokyo, Japan), and the long-chain fatty acids were subjected to esterification using a mixture of 2 mol/L KOH/CH_3_OH. Subsequently, the fatty acid content was analyzed using GC-MS (456-GC; BRUKER, Ltd., Billerica, Germany). The injector and detector temperatures were set at 220 °C and 245 °C, respectively. A capillary column (flexible quartz capillary column: 30 m × 0.25 mm × 0.25 μm) was employed, with an initial temperature of 40 °C, increasing at a rate of 10 °C per minute until it reached 240 °C. The peak areas or heights were calculated, and these data were subsequently utilized for concentration determination and quantitative analysis [15].

### 2.7. Statistical Analysis

The statistical analysis was performed using SPSS 21.0 (SPSS Inc., Chicago, IL, USA) and Origin 9.0 (Origin Lab Corp., Northampton, MA, USA). All data represent the results of six independent replicates. One-way analysis of variance (ANOVA) was conducted, and Tukey’s test was used for a post hoc comparison of means. Significance was determined at *p* < 0.05, and differences were considered highly significant if *p* < 0.01.

## 3. Results

### 3.1. Analysis of Microbial Community in Silage

**Figure 1:**
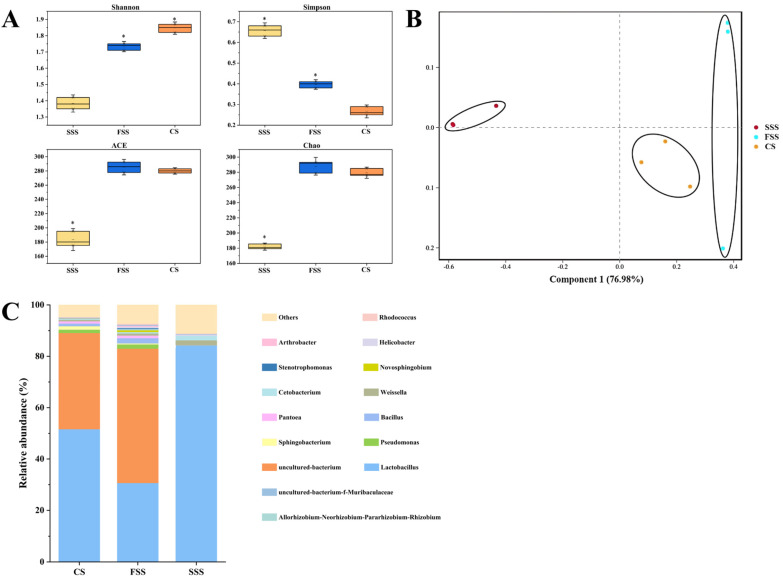
Bacterial community diversities of silage. (**A**) Alpha diversity of bacterial community. (**B**) Principal coordinates analysis plots with unweighted Unifrac dissimilarity of bacterial community. (**C**) Relative abundance of bacterial community at genus level in silage.* Means were significantly different in comparison with the CON group (* *p* < 0.05). SSS, sugar sweet sorghum silage; FSS, forage sweet sorghum silage; CS, corn silage.

The alpha diversity analysis of microbial communities in SSS, FSS, and CS is shown in Figure 1A, with a total of 303 to 329 OTUs detected. Based on 97% species similarity, 533557 high-quality 16S rRNA sequences were clustered into 434 OTUs. The coverage of all samples was above 0.99, indicating that most bacteria were detected. Compared with CS, the Shannon index was significantly decreased, while the Simpson index was significantly increased in SSS and FSS. SSS also significantly reduced the Ace and Chao indices. Therefore, the microbial community diversity in SSS and FSS is lower than that of CS.

Principal component analysis (PCoA) of the bacterial communities is shown in Figure 1B, where component 1 and component 2 explain 76.98% and 6.17% of the total variance, respectively. SSS was clearly separated from FSS and CS. Therefore, there are differences in the microbial composition and structure of SSS, FSS, and CS.

The relative abundance of silage bacteria at the genus level is shown in Figure 1C. The dominant genera in CS were *Lactobacillus* (51.64%), followed by uncultured _bacterium (37.48%) and *Pseudomonas* (1.31%), while others accounted 4.48% of total microbial community. The FSS was dominated by uncultured bacterium (52.31%), *Lactobacillus* (30.68%), and *Pseudomonas* (1.68%). The highest relative abundance of uncultured bacterium (52.31%) was detected in FSS. The SSS was dominated by *Lactobacillus* (82.23%) and *Pantoea* (0.26%), followed by *Weissella* (0.206%) and *Cetobacterium* (0.205%). After ensiling, although *Lactobacillus* were the dominant microbes in silage samples, their relative abundances varied with each other. Thus, the variation in the microbial communities in silage might be one critical factor leading to differences in animal feeding and meat quality.

### 3.2. Goat Growth Performance

**Figure 2:**
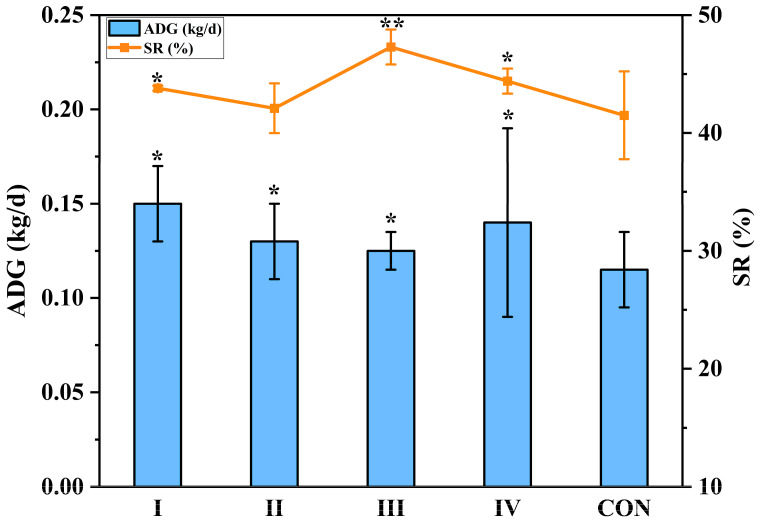
Goat performance of different groups. Error bars represent the mean standard deviation. * Means were significantly different in comparison with the CON group (* *p* < 0.05; ** *p* < 0.01). I, 50% FSS; II, 70% FSS; III, 50% SSS; IV, 70% SSS; CON, 50% CS; (FSS, forage sweet sorghum silage; SSS, sugar sweet sorghum silage; CS, corn silage).

Goat performance for this study, including the average daily gain (ADG) and slaughter rate (SR) of the goats, is presented in Figure 2. The ADG of the experimental groups were higher than that of the CON group. In addition, the ADG of the groups I and IV were, respectively, increased by 27.6% and 21.6% (*p* < 0.05) compared with the CON group. Group III has the highest SR at 47.3%, which was significantly increased by 12.4% (*p* < 0.01) compared with the CON group. The SR in group I and group IV were significantly increased by 5.5% (*p* < 0.05) and 6.9% (*p* < 0.05) compared with the CON group, respectively. Therefore, compared with CS, both SSS and FSS have a positive effect on increasing the ADG and SR of goats. There is no significant difference in the ADG of goats among different experimental groups, and the SR of experimental group III is significantly higher than that of other treatment groups; therefore, 50% SSS is the most effective for improving the growth performance of goats.

### 3.3. Goat Meat Quality

The results for the pH, initial moisture, ash, crude fat and protein (N × 6.38) of goat meat are shown in Table 3. The CP contents of group III and group IV were higher than that of CON group, which were increased by 24.7% and 24.2%, respectively. Compared with the CON group, the crude fat (CF) content (5.02 ± 0.11) in the goat meat of group III was increased by 5.0%, which showed that group III of the SSS formula can increase the fat content. Except for this, in this trial, there was no significant difference in the levels of CP and CF between other experimental groups and the CON group. Moreover, there was no significant difference in the pH, ash content, and initial moisture between the experimental and CON group. It may be that the change in raw feed stuff has no significant effect on these contents in meat samples.

### 3.4. The Fatty Acid Composition in Meat

As can be seen from Table 4, 29 kinds of fatty acids in this study were detected. The levels of SFA, MUFA, and PUFA were approximately 50%, 40%, and 10%, respectively, while the levels of UFA ranged from 41.44% to 47.90%.The contents of unsaturated fatty acids (UFA) in group I, group II and group III were increased after the 90 d feeding experiment and were, respectively, increased by 12.7%, 15.6%, and 11.9% (*p* < 0.05) compared with the CON group, and the greatest differences were in the C18:1(n-9 cis) content, which were, respectively, higher by 15.5%, 18.4%, and 19.9% in group I, group II, and group II than that in the CON group (*p* < 0.05). The ratios of UFA and SFA of each experimental groups were higher than in the CON group, especially in group I and group II, being significantly higher than that in the CON group (*p* < 0.01), which reached 0.94, and the ratio in group III was also significantly higher than that in the CON group (*p* < 0.05), which reached 0.88. Regarding PUFA, except for the C20:4 content of group II, other contents are not significantly different in the control and experimental groups. All in all, the content of unsaturated fatty acids (UFA) and the ratio of UFA/SFA of group I and group II with FSS and group III with 50% SSS were significantly increased by the substitution of SS diets for the standard CS diet in the present study.

### 3.5. Comparison of Amino Acids in Meat

Proteins are constituted of about 20 kinds of amino acid (AA), including eight essential amino acids (EAA). Among those amino acids, Asparagine (Asn) and Glutamine (Glu) can compound with common foods. Eighteen kinds of amino acids in meat in this study were analyzed, and the results represented in Table 5. The total essential amino acid content in group I and group II was higher (*p* < 0.05) than in the control group, increased by 6.33% and 9.55%, respectively. The content of total nonessential amino acids (NEAA) in group II was higher (*p* < 0.05) than in the control group, increased by 6%. Compared with the CON group, the contents of Methionine (Met) in group II and group IV were increased by 933.33% (*p* < 0.01) and 944.4% (*p* < 0.01), respectively. The percentages of aspartic acid (Asp), which was classified as umami, were higher in group I (7.13%) and group II (7.21%) than the control group (6.99%), while the Asp contents of group III (5.60%) and group IV (5.66%) were lower than the control group. As for proline (Pro) which was classified as sweet, in group I (5.74%), group II (5.84%), and group IV (5.86%), the contents were significantly (*p* < 0.05) higher than in the control (5.34%). In general, the amounts of total AA of group I (74.84%) and group II (76.83%) were greater (*p* < 0.05) than those of the control group (71.54%). Hence, the flavors of group I and group II in this trial contained more sweetness, umami, and nutrition than the CON group.

### 3.6. Analysis of Microbial Community in the Rumen of Boer Goats

**Figure 3:**
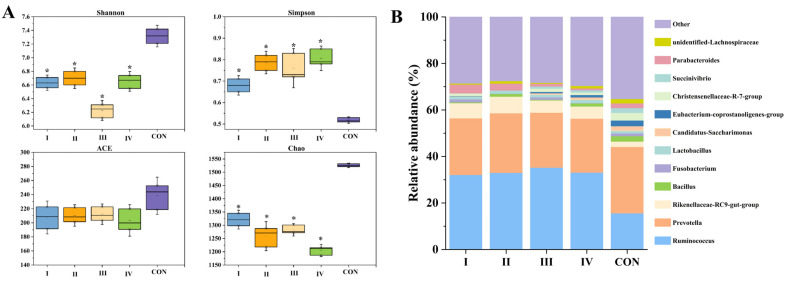
Bacterial community diversities of silage. (**A**) Alpha diversity of bacterial community. (**B**) Relative abundance of bacterial community at genus level in rumen of Boer goats. * Means were significantly different in comparison with the CON group (* *p* < 0.05). I, 50% FSS; II, 70% FSS; III, 50% SSS; IV, 70% SSS; CON, 50% CS; (FSS, forage sweet sorghum silage; SSS, sugar sweet sorghum silage; CS, corn silage).

The diversity of microbial communities in the rumen of Boer goats was analyzed after feeding with different dietary formulations, with a total of 1205 to 1379 OTUs detected. The coverage values of all samples were above 0.99, indicating that most of the bacteria were detected. The α-diversity analysis of the rumen microbial community (Figure 3A) showed that compared to feeding with CS, SSS and FSS increased the Simpson index and decreased the Shannon and Chao indices. This suggests that feeding SSS and FSS affected the composition of the rumen microbial community, leading to a decrease in species diversity and an increase in the abundance of dominant microorganisms.

The bacterial community composition at the genus level in the rumen of Boer goats after feeding with different dietary formulations is shown in Figure 3B. The dominant genera in different groups were *Ruminococcus*, *Prevotella*, and *Rikenellaceae-RC9-gut-group*. In the CON treatment group, the relative abundances of *Ruminococcus* and *Prevotella* were 15.63% and 28.56%, respectively. However, feeding Boer goats with diets containing SSS and FSS increased the relative abundance of *Ruminococcus*, which reached 32.12%, 33.02%, 35.26%, and 33.12% in groups I, II, III, and IV, respectively. On the other hand, the relative abundance of *Prevotella* decreased in groups I, II, III, and IV, with values of 24.36%, 25.63%, 23.63%, and 23.21%, respectively. The abundance of Rikenellaceae_RC9 did not show a significant trend across different treatment groups, but its abundance ranked third after *Ruminococcus* and *Prevotella*, with a relative abundance ranging from 2.31% to 7.13%. The relative abundance of other genera is low, and their contribution to the microbial community composition and structure is minimal.

## 4. Discussion

### 4.1. Chemical Composition and Microbial Community of Silage

Through the analysis of silage characteristics, it was found that compared to CS, SSS and FSS have higher levels of NDF, ADF, and CF, and this result is consistent with previous studies [16,17]. Additionally, SSS and FSS also exhibited higher levels of WSCs. The WSC content is likely related to the WSC content of the crop before ensiling [18], and the addition of silage inoculants may promote the degradation and conversion of structural carbohydrates, thereby increasing the WSC content [19]. Compared to FSS, SSS showed higher DM, NDF, ADF, and CF contents, and the relative abundance of Lactobacillus in SSS reached 82.23%, while the relative abundance in CS and FSS was only 51.64% and 30.68%, respectively. Lactobacillus is known for its strong acid tolerance and is typically active in the later stages of the silage process [20]. It is usually associated with a good fermentation process in silage, and the increased abundance of Lactobacillus helps improve the fermentation quality of silage [14]. Thus, the chemical and microbiological compositions reflect the nutrient content of the silage as well as the state of silage fermentation.

### 4.2. Growth Performance and Rumen Microbiological Analysis in Goats

Goat meat performance is highly dependent on the feed composition. The results showed that both SSS and FSS significantly (*p* < 0.05) improved the ADG and SR compared to the CON group. This was due to silage characteristics leading to differences in the ADG and SR. Compared to CS, SSS and FSS had higher DM and water-soluble carbohydrate (WSC) contents, providing a quicker source of energy for the goats [21]. The low protein content and digestibility of CS may be the main reasons for the lower weight gain in goats [22]. Feeding FSS and SSS diets also caused significant changes in the rumen microbial community, reducing the microbial diversity and altering its composition. *Ruminococcaceae*, a major fiber-degrading bacterium in the rumen, was increased in the treatment groups fed FSS and SSS. *Ruminococcaceae* produces cellulases, hemicellulases, and xylanases, which break down cellulose and hemicellulose in roughage, facilitating better nutrient availability [23]. Additionally, the high levels of neutral detergent fiber (NDF) in FSS and SSS require degradation in the rumen before being absorbed by the animals. The increased abundance of *Ruminococcaceae* helps convert these structural carbohydrates into nutrients that can be utilized for ruminant growth, providing more energy. *Prevotellaceae,* another dominant family in the rumen microbiota, is also important for the degradation and utilization of starch and plant cell wall polysaccharides like xylan and pectin [24,25]. In this study, *Prevotellaceae* was found to be the dominant genus in the rumen of goats fed all silage types, with the highest levels in the corn silage (CON) group. CS has a higher starch content compared to SSS and FSS, which is the main reason for the increased abundance of *Prevotellaceae*. *Ruminococcaceae* and *Prevotellaceae* belong to the phyla Firmicutes and Bacteroidetes, respectively. The increase in the relative abundance of *Ruminococcaceae* may lead to a higher Firmicutes/Bacteroidetes ratio, which is associated with enhanced fat accumulation [26]. In conclusion, feeding FSS and SSS to Boer goats not only improved their growth performance but also altered the rumen microbial community in ways that favor better nutrient utilization, particularly through the breakdown of fibrous plant materials, thus increasing the ADG and SR.

### 4.3. Goat Meat Quality

The anaerobic fermentation of muscle glycogen to produce lactic acid and the catabolism of ATP to produce phosphate ions in the muscles of slaughtered animals cause a decrease in pH [27,28,29]. Meat is considered fresh at pH 5.8 to 6.2 and spoiled when the pH is above 6.5 [30]. In our experiment, all treatment groups were within this range of pH and were not significantly different (*p* > 0.05). The natural moisture content of meat is about 70% but varies according to feed, type, and muscle cut [31]. The moisture content of goat meat in all treatment groups ranged from 77.4 to 78.75%. Higher crude protein content in meat represents better nutritional value, while fat content is an important parameter that affects the sensory characteristics of meat, especially the tenderness and flavor of the meat [32]. The crude protein and crude fat contents were significantly increased in group III containing 50% SSS. Thus, SSS had a positive effect on the nutritional value of the meat.

### 4.4. Fatty Acids in Goat Meat 

The composition of fatty acids has an important influence on the quality of meat. Saturated fatty acid (SFA) intake increases low density lipoprotein cholesterol in the blood, which is associated with an increased risk of cardiovascular diseases [33]. The SFA content of the meat in group I was significantly lower than that of CON (*p* < 0.05), while there was no significant difference in the other experimental groups (*p* > 0.05). UFA of the right type and composition had a significant effect on reducing cholesterol and blood fibrinogen [34,35]. The results for unsaturated fatty acids (UFA) in group I, group II, and group III were significantly higher than those in the CON group, with increases of 12.7%, 15.6%, and 11.9%, respectively (*p* < 0.05). Many studies with ruminants show that different nutritional conditions can change the muscle lipid fatty acid composition, polyunsaturated fatty acids (PUFA) level, and n-3:n-6 PUFA ratio [36]. As for PUFA, especially arachidonic acid (C20:4 n-6), linoleic acid (C18:2 n-6), and linolenic acid (C18:3 n-3), they were found to have particular benefits on human health. And the results of the present study have proven the assumption that MUFA were obtained based on differences in the feed ingredients. FSS and SSS, which differed from the CS component, significantly increased the content of C18:1(n-9 cis). The C18:1(n-9 cis) contents in group I, group II, and group III were, respectively, higher by 15.5%, 18.4%, and 19.9% than that in the CON group (*p* < 0.05).

### 4.5. Amino Acids in Goat Meat

Protein is one of the most important chemical components of meat, and amino acids are the basic building blocks of proteins. Amino acids are essential nutrients for animals, and their composition and levels directly affect the quality of meat in livestock and poultry. The flavor of goat meat is considered the most important factor influencing consumer acceptance [37]. Compared to the CON group, 50% and 70% FSS diets increases the contents of essential amino acids such as Val, Met, Ile, Leu, Lys, and Arg. Among these, Leu is a functional amino acid that plays a regulatory role in extrahepatic metabolism [38]. Su, Xu [39] showed that supplementing leucine in the diet has beneficial effects on mitochondrial biogenesis and energy metabolism in the livers of pigs. Arg and Lys are associated with the umami and sweetness of meat, respectively, and also contribute to increasing the content of the nonessential amino acid Pro, which is also related to sweetness. Thus, feeding FSS increased the content of essential and flavor-related amino acids in meat, which was mainly related to the NDF content in silage and rumen microorganism. FSS has a higher NDF content than that of CS. However, feeding FSS, as compared to CS, increases the abundance of *Ruminococcaceae*, which degrade NDF. At the same time, the NDF of FSS was lower and more readily degraded by rumen microorganisms compared to SSS. Therefore, volatile fatty acids (e.g., lactic acid and acetic acid) released during NDF degradation provide an adequate carbon source for microbial synthesis of proteins, which indirectly promotes the production of amino acids. Compared to the CON group, a 50% SSS diet does not show significant differences in amino acids, and a 70% SSS diet can increase the content of gly and pro, which has a positive impact on meat flavor. The results suggest that the effect of SSS on amino acids was related to the amount added. This may be due to the low SSS addition. Lv, Chen [17] showed that the levels of ser, Ile, Phe, Pro, and total amino acids increase linearly as the proportion of sweet sorghum increases, replacing corn silage with sweet sorghum silage (*p* < 0.05). Therefore, the addition of FSS and SSS to the diet had a positive effect on increasing the amino acid content and improving the flavor of the meat.

In general, this study demonstrated the impact of different types, proportions, and compositions of silage on goat meat quality. The results support the feasibility of replacing corn silage with sweet sorghum silage in Boer goat feeding, which can improve both goat body weight and meat quality. However, the sample size in this study is relatively small, and therefore, the results need to be further validated in studies with larger sample sizes to ensure their broader applicability and reliability

## 5. Conclusions

In this study, CS was replaced with SSS and FSS as feed sources for Boer goats. SSS and FSS contained higher WSC and CP contents and, when fed, led to changes in goat rumen microbes, increased the relative abundance of *Ruminococcaceae*, promoted the degradation of NDF, provided an adequate source of nutrients for the goats, and increased the ADG and SR. FSS in the diets increased the levels of EAAs and flavor-related amino acids, positively influencing the flavor of the meat. Meanwhile, SSS in diets was able to increase the crude protein and crude fat contents in goat meat, improving its tenderness and nutritional value. Therefore, we suggest that diets incorporating SS in the place of CS can improve both the growth performance and meat quality of Boer goats when available.

## Figures and Tables

**Table 1:** The chemical composition of the silage.

Item	Silage		
	Forage Sweet Sorghum Silage (FSS)	Sugar Sweet Sorghum Silage (SSS)	Whole-Corp Corn Silage (CS)
DM, %	32.98	51.71	28.31
pH	4.59	5.08	4.78
WSCs, % DM	22.35	17.16	10.56
CP, % DM	5.76	5.87	5.18
NDF, % DM	75.98	83.93	63.58
ADF, % DM	46.62	55.86	44.71
Crude fiber, % DM	40.14	47.32	30.5
Ash, % DM	8.45	7.34	8.08
Lactic acid, g/kg DM	10.03	8.32	8.5
Acetic acid, g/kg DM	2.30	2.18	2.34
Propionic acid, g/kg DM	0.87	1.01	1.18

DM, dry matter; WSC, water-soluble carbohydrates; CP, crude protein; NDF, neutral detergent fiber; ADF, acid detergent fiber.

**Table 2:** Diet composition of the five treatments (% of DM).

Item	Silage				
	Group I	Group II	Group III	Group IV	CON
Concentrate diet					
Ingredient, % of diet DM					
Maize, %	64.0	64.0	64.0	64.0	64.0
Soybean meal, %	15.0	15.0	15.0	15.0	15.0
Wheat bran, %	15.0	15.0	15.0	15.0	15.0
Salt, %	1.0	1.0	1.0	1.0	1.0
Premix, %	5.0	5.0	5.0	5.0	5.0
Roughage diet					
Ingredient, % of diet FM					
Forage sweet sorghum silage, %	50.0	70.0	-	-	-
Sugar sweet sorghum silage, %	-	-	50.0	70.0	-
Corn silage, %	-	-	-	-	50.0
Wheat stalk, %	30.0	10.0	30.0	10.0	30.0
Alfalfa, %	20.0	20.0	20.0	20.0	20.0

DM, dry matter; FM, fresh matter. Group I, 50% FSS; Group II, 70% FSS; Group III, 50% SSS; Group IV, 70% SSS; CON, 50% CS (FSS, forage sweet sorghum silage; SSS, sugar sweet sorghum silage; CS, corn silage).

**Table 3:** Meat quality of goats in different groups.

Item	Treatment				
	Group I	Group II	Group III	Group IV	CON
pH	6.08 ± 0.14	6.00 ± 0.27	5.80 ± 0.21	5.90 ± 0.16	5.86 ± 0.09
Moisture, %	77.40 ± 1.56	78.75 ± 2.33	77.5 ± 2.97	77.4 ± 0.57	76.45 ± 1.34
CP, %	23.44 ± 2.93	23.20 ± 2.76	24.66 ± 3.03 *	24.21 ± 2.31*	22.86 ± 3.39
Crude fat, %	4.54 ± 0.23	4.62 ± 0.12	5.02 ± 0.11 *	4.96 ± 0.21	4.52 ± 0.18
Ash, %	1.08 ± 0.0020	1.00 ± 0.0010	1.04 ± 0.0004	0.96 ± 0.0001	1.01 ± 0.0006

* Means were significantly different in comparison with the CON group (* *p* < 0.05). Group I, 50% FSS; Group II, 70% FSS; Group III, 50% SSS; Group IV, 70% SSS; CON, 50% CS; (FSS, forage sweet sorghum silage; SSS, sugar sweet sorghum silage; CS, corn silage).

**Table 4:** The main fatty acids contained in different groups of goat meat (%).

Item	Treatment				
	Group I	Group II	Group III	Group IV	CON
C6:0	0.22 ± 0.07	0.21 ± 0.01	0.23 ± 0.02	0.22 ± 0.03	0.24 ± 0.11
C10:0	0.17 ± 0.04	0. 15 ± 0.02	0.14 ± 0.03	0.16 ± 0.01	0.18 ± 0.02
C12:0	0.23 ± 0.67	0.01 ± 0.00	0.03 ± 0.02	0.27 ± 0.02	0.29 ± 0.12
C13:0	0.09 ± 0.02	0.10 ± 0.01	0.09 ± 0.01	0.09 ± 0.05	0.10 ± 0.03
C14:0	0.45 ± 0.68	0.43 ± 0.42	0.47 ± 0.23	0.42 ± 0.23	0.47 ± 0.23
C15:0	0.21 ± 0.08	0.19 ± 0.03	0.20 ± 0.01	0.20 ± 0.01	0.21 ± 0.03
C16:0	25.40 ± 1.51	26.42 ± 1.67	27.14 ± 1.76	27.56 ± 1.68	30.36 ± 1.81
C17:0	0.16 ± 0.06	0.16 ± 0.04	0.12 ± 0.01	0.14 ± 0.03	0.13 ± 0.02
C18:0	21.91 ± 1.41	22.23 ± 1.68	23.01 ± 1.59	23.31 ± 1.57	22.21 ± 1.41
C20:0	0.77 ± 0.48	0.79 ± 0.77	0.67 ± 0.23	0.80 ± 0.23	0.79 ± 0.23
C21:0	0.12 ± 0.04	0.11 ± 0.01	0.11 ± 0.02	0.10 ± 0.03	0.10 ± 0.03
C22:0	0.23 ± 0.65	0.25 ± 0.46	0.29 ± 0.19	0.28 ± 0.03	0.28 ± 0.21
C23:0	0.01 ± 0.00	0.02 ± 0.00	0.01 ± 0.00	0.02 ± 0.00	0.01 ± 0.00
C16:1	6.83 ± 0.60	7.75 ± 1.02	6.87 ± 0.57	6.70 ± 1.20	6.26 ± 1.23
C18:1 n-9 trans	1.23 ± 0.51	1.22 ± 0.84	1.24 ± 0.81	1.23 ± 0.81	1.22 ± 0.79
C18:1 n-9 cis	28.69 ± 1.34 *	29.41 ± 1.96 *	29.31 ± 1.52 *	26.70 ± 1.41	24.84 ± 1.07
C20:1	0.11 ± 0.04	0.12 ± 0.02	0.13 ± 0.03	0.11 ± 0.02	0.13 ± 0.04
C22:1	0.02 ± 0.00	0.02 ± 0.00	0.03 ± 0.01	0.02 ± 0.00	0.02 ± 0.00
C18:2 n-6	2.53 ± 0.11	2.85 ± 0.09	2.79 ± 0.10	2.60 ± 0.10	2.54 ± 0.10
C18:3 n-6	0.02 ± 0.00	0.03 ± 0.01	0.02 ± 0.00	0.02 ± 0.00	0.03 ± 0.00
C20:3 n-6	0.12 ± 0.02	0.11 ± 0.01	0.12 ± 0.03	0.12 ± 0.02	0.11 ± 0.02
C20:4 n-6	4.37 ± 0.67	4.78 ± 0.23 *	4.47 ± 0.23	3.98 ± 0.48	3.82 ± 1.01
C22:4 n-6	0.02 ± 0.00	0.02 ± 0.00	0.03 ± 0.01	0.03 ± 0.00	0.03 ± 0.01
C18:3 n-3	1.95 ± 0.23	0.78 ± 0.48	0.55 ± 0.42	1.68 ± 0.41	1.60 ± 0.39
C20:3 n-3	0.03 ± 0.00	0.03 ± 0.00	0.04 ± 0.01	0.05 ± 0.01	0.04 ± 0.00
C20:5 n-3	0.21 ± 0.01	0.22 ± 0.04	0.22 ± 0.01	0.22 ± 0.01	0.21 ± 0.03
C22:5 n-3	0.31 ± 0.07	0. 30 ± 0.07	0.29 ± 0.06	0.29 ± 0.08	0.31 ± 0.07
C22:6 n-3	0.17 ± 0.03	0.16 ± 0.02	0.17 ± 0.03	0.18 ± 0.03	0.18 ± 0.02
SFA	49.97 ± 1.63 *	51.07 ± 1.10	52.51 ± 1.04	53.57 ± 2.02	55.37 ± 2.11
MUFA	36.88 ± 1.95	38.52 ± 2.01 *	37.59 ± 1.84	34.76 ± 1.68	32.47 ± 1.51
PUFA	9.82 ± 0.21 *	9.38 ± 1.18	8.78 ± 1.86	9.25 ± 1.23	8.97 ± 1.08
UFA	46.70 ± 1.65 *	47.90 ± 1.14 *	46.36 ± 1.43 *	44.01 ± 1.52	41.44 ± 1.37
UFA/SFA	0.935 ± 0.02 **	0.938 ± 0.01 **	0.883 ± 0.01*	0.822 ± 0.02	0.748 ± 0.01

SFA, saturated fatty acids; SD, standard error of the means. MUFA, monounsaturated fatty acids; PUFA, polyunsaturated fatty acids; UFA, unsaturated fatty acids. SD—standard error of the means. *^,^ ** Means were significantly different in comparison with the CON group (* *p* < 0.05; ** *p* < 0.01). Group I, 50% FSS; Group II, 70% FSS; Group III, 50% SSS; Group IV, 70% SSS; CON, 50% CS; (FSS, forage sweet sorghum silage; SSS, sugar sweet sorghum silage; CS, corn silage).

**Table 5:** The amounts of 18 types of amino acids of protein in different groups.

Item	Treatment				
	Group I	Group II	Group III	Group IV	CON
Thr	3.60 ± 0.03	3.71 ± 0.02	2.83 ± 0.01	2.76 ± 0.01	3.50 ± 0.02
Val	3.90 ± 0.01 *	3.89 ± 0.2 *	2.98 ± 0.01	3.04 ± 0.01	3.64 ± 0.03
Met	0.23 ± 0.00	0.93 ± 0.01 **	0.11 ± 0.01	0.94 ± 0.01 **	0.09 ± 0.00
Ile	3.62 ± 0.02 *	3.61 ± 0.01 *	2.76 ± 0.01	2.67 ± 0.01	3.34 ± 0.03
Leu	6.24 ± 0.17 *	6.30 ± 0.13 *	4.86 ± 0.09	4.85 ± 0.08	5.93 ± 0.11
Phe	3.00 ± 0.05	3.05 ± 0.04	2.68 ± 0.02	2.40 ± 0.01	2.88 ± 0.03
Lys	6.35 ± 0.13	6.43 ± 0.13 *	4.86 ± 0.03	4.84 ± 0.02	6.02 ± 0.13
Trp	1.11 ± 0.04 *	0.98 ± 0.02	0.68 ± 0.01 *	0.81 ± 0.01	0.98 ± 0.02
Asp	7.13 ± 0.17	7.21 ± 0.15	5.60 ± 0.11	5.66 ± 0.09	6.99 ± 0.12
Ser	2.96 ± 0.01	3.04 ± 0.03	2.40 ± 0.02 *	2.39 ± 0.01 *	2.86 ± 0.03
Glu	13.02 ± 0.21	13.48 ± 0.23	10.62 ± 0.21	10.68 ± 0.22	12.90 ± 0.27
Pro	5.74 ± 0.12 *	5.84 ± 0.11 *	5.19 ± 0.16	5.86 ± 0. 17 *	5.34 ± 0.16
Gly	3.18 ± 0.02	3.30 ± 0.04	2.82 ± 0.01	3.81 ± 0.05 *	2.97 ± 0.02
Ala	4.41 ± 0.15	4.44 ± 0.13	3.55 ± 0.11	3.93 ± 0.09	4.20 ± 0.02
Tyr	2.43 ± 0.02	2.39 ± 0.03	1.76 ± 0.05	1.84 ± 0.04	2.06 ± 0.04
His	2.34 ± 0.01	2.22 ± 0.02	1.59 ± 0.02	1.63 ± 0.01	1.95 ± 0.03
Arg	4.69 ± 0.12	4.81 ± 0.13 *	3.74 ± 0.11	4.00 ± 0.12 *	4.55 ± 0.15
Cys	2.00 ± 0.01	2.18 ± 0.02	1.52 ± 0.00	1.93 ± 0.01	2.32 ± 0.02
EAA	28.05 ± 0.67 *	28.90 ± 0.47 *	21.76 ± 0.65	22.31 ± 0.47	26.38 ± 0.42
NEAA	47.90 ± 1.85	48.91 ± 1.42 *	38.79 ± 1.21	41.73 ± 1.54	46.14 ± 1.35
Total	74.84 ± 2.21 *	76.83 ± 2.31 *	59.87 ± 2.01	63.23 ± 2.43	71.54 ± 2.56

EAA—essential amino acids; NEAA—nonessential amino acids. SD—standard error of the means. *^,^ ** Means were significantly different in comparison with the CON group (* *p* < 0.05; ** *p* < 0.01). Group I, 50% FSS; Group II, 70% FSS; Group III, 50% SSS; Group IV, 70% SSS; CON, 50% CS; (FSS, forage sweet sorghum silage; SSS, sugar sweet sorghum silage; CS, corn silage).

## Data Availability

The original contributions presented in this study are included in the article. Further inquiries can be directed to the corresponding authors.
